# Early versus delayed initiation of antiretroviral therapy for Indian HIV-Infected individuals with tuberculosis on antituberculosis treatment

**DOI:** 10.1186/1471-2334-12-168

**Published:** 2012-07-31

**Authors:** Sanjeev Sinha, Rahul C Shekhar, Gurjeet Singh, Nipam Shah, Hafiz Ahmad, Narendra Kumar, Surendra K Sharma, JC Samantaray, Sanjai Ranjan, Meera Ekka, Vishnu Sreenivas, Ronald T Mitsuyasu

**Affiliations:** 1 Department of Medicine, All India Institute of Medical Sciences, Ansari Nagar, New Delhi, 110029, India; 2 Department of Microbiology, All India Institute of Medical Sciences, New Delhi, India; 3 Department of Biostatistics, All India Institute of Medical Sciences, New Delhi, India; 4 UCLA Center for Clinical AIDS Research & Education, University of California, Los Angeles, USA

**Keywords:** Antiretroviral, Early, Delayed, HIV, Tuberculosis

## Abstract

**Background:**

For antiretroviral therapy (ART) naive human immunodeficiency virus (HIV) infected adults suffering from tuberculosis (TB), there is uncertainty about the optimal time to initiate highly active antiretroviral therapy (HAART) after starting antituberculosis treatment (ATT), in order to minimize mortality, HIV disease progression, and adverse events.

**Methods:**

In a randomized, open label trial at All India Institute of Medical Sciences, New Delhi, India, eligible HIV positive individuals with a diagnosis of TB were randomly assigned to receive HAART after 2-4 or 8-12 weeks of starting ATT, and were followed for 12 months after HAART initiation. Participants received directly observed therapy short course (DOTS) for TB, and an antiretroviral regimen comprising stavudine or zidovudine, lamivudine, and efavirenz. Primary end points were death from any cause, and progression of HIV disease marked by failure of ART.

**Findings:**

A total of 150 patients with HIV and TB were initiated on HAART: 88 received it after 2-4 weeks (early ART) and 62 after 8-12 weeks (delayed ART) of starting ATT. There was no significant difference in mortality between the groups after the introduction of HAART. However, incidence of ART failure was 31% in delayed versus 16% in early ART arm (p = 0.045). Kaplan Meier disease progression free survival at 12 months was 79% for early versus 64% for the delayed ART arm (p = 0.05). Rates of adverse events were similar.

**Interpretation:**

Early initiation of HAART for patients with HIV and TB significantly decreases incidence of HIV disease progression and has good tolerability.

**Trial registration:**

CTRI/2011/12/002260

## Background

According to 2010 report of Joint United Nations Programme on HIV and AIDS (UNAIDS), the global burden of human immunodeficiency virus (HIV) infection was 33.3 million in 2009, with 2.6 million incident cases and 1.8 million acquired immune deficiency syndrome (AIDS) related deaths [1]. Tuberculosis (TB) in HIV-positive patients is one of the leading causes of morbidity and mortality, and therefore, a major constraint in the fight against HIV. Approximately 30% of HIV-infected persons all over the world are estimated to have latent TB infection [2]. World Health Organization (WHO) reported in 2010 that HIV-positive population experienced 11-13% of 9.4 million incident TB cases worldwide, and 0.38 million TB related deaths during the previous year [3]. TB is responsible for approximately one in four deaths in HIV patients globally [4].

With about one fifth of the global burden, TB continues to be a public health challenge in India. About 40% of Indian population is supposed to be infected with TB bacillus. The disease is responsible for 17.6% of deaths from communicable diseases and for 3.5% of all causes of mortality [5]. Even though the endemic is maintained predominantly by non HIV TB cases, the situation gets complicated by India being the third highest HIV burdened country. As per 2009-2010 report from National AIDS Control Organization (NACO), the prevalence of HIV infection by the end of 2008 was 0.29% in Indian adults [6]. However, among TB patients in the country, HIV prevalence was as high as 6.7% in the same year according to WHO estimates. About 4.85% of incident TB cases in India were HIV positive in 2007 [5].

It is well established that HIV increases the risk for TB (acquisition, reactivation and reinfection), alters its clinical presentation, and reduces survival compared to patients with TB and no HIV infection [7-9]. With introduction of timely ART, the incidence of new TB infection can also be reduced by up to 90% [10]. However, the question is when to start highly active antiretroviral therapy (HAART) in a patient with HIV and TB (HIV-TB) who is taking treatment for tuberculosis. On one hand, early initiation of HAART has the potential benefit of reducing mortality and morbidity among recipients, on the other, physicians tend to defer it during TB treatment because of concerns that concomitant antituberculosis and antiretroviral treatment may result in overlapping toxicities of drugs, low adherence to medications due to high pill burden, and higher risk of paradoxical worsening of signs/symptoms of TB that is called immune reconstitution inflammatory syndrome (IRIS) [11]. These factors impart considerable uncertainty to the optimal timing of HAART initiation after starting TB treatment.

There have been several studies that deal with the question of timing of HAART commencement in the setting of TB treatment in order to gain optimal outcome. Complete results of some recently concluded randomized controlled trials that address the issue are yet to come out [12,13]. Some of the published studies have limitations like small sample size or lack of a control group, while a few others are primarily observational or retrospective in nature [12]. This paper presents the findings of a randomized controlled clinical trial that was conducted to compare early (2-4 weeks) and delayed (8-12 weeks) initiation of antiretroviral therapy after commencement of antituberculosis treatment in North Indian HIV-infected adults with tuberculosis, in terms of outcomes like mortality and HIV disease progression.

## Methods

### Trial design

This randomized open-label trial was conducted at All India Institute of Medical Sciences (AIIMS), a tertiary referral centre in New Delhi, India. ART-naïve individuals with HIV and recently diagnosed TB were enrolled and randomized into one of the two arms of the study using one to one allocation ratio. One arm was scheduled to receive early HAART, starting within 2-4 weeks of commencement of antituberculosis treatment (ATT), and the other arm was to have delayed HAART at 8-12 weeks after starting ATT. For TB, patients were categorized according to Indian Revised National Tuberculosis Control Programme (RNTCP) guidelines for thrice weekly directly observed treatment short-course (DOTS) [6], and treated accordingly with free drugs provided by RNTCP. Antiretroviral drugs were provided free of cost by NACO as part of the National AIDS Control Programme (NACP). Patients were followed for 12 months after HAART initiation. Ethics approval for the study was obtained from the AIIMS institutional ethics committee.

### Participants

All ART naïve HIV positive patients with active TB presenting in the hospital’s ART Centre, DOTS Centre, infectious diseases and chest clinics, medical OPD, and wards were screened for eligibility. Subjects aged over 18 years, who had seven or fewer days of cumulative previous ART (unless taken during pregnancy to prevent mother-to-child-transmission), and who had not started ATT or received less than 14 days of the same, were eligible for entry into this study. Both confirmed and probable diagnoses of TB were permitted for inclusion. Confirmed cases included those with positive smear (using Ziehl Neelsen staining) for acid fast bacilli, and/or culture growth (on solid Lowenstein Jensen medium). As per WHO guidelines, probable diagnosis is based on the clinician’s judgment where acid-fast bacilli are not demonstrable but sufficient clinical suspicion and/or radiological evidence exists to initiate empiric TB therapy [14]. Liver function tests, i.e. SGOT, SGPT, and serum bilirubin, within five times the upper limit of normal range, and for female subjects, a negative urine pregnancy test were required for study entry. Exclusion criteria were pregnancy, concomitant diabetes mellitus, epilepsy, severe illness (e.g. loss of consciousness, severe hempotysis etc.), and multi-drug resistant TB (MDR TB). MDR TB was excluded by a positive history of the same. Besides, all sputum samples positive for *Mycobacterium tuberculosis* were further screened using radiometric BACTEC 460 TB system.

### Interventions

Patients who satisfied the screening criteria and provided written informed consent had their HIV status confirmed using Enzyme-linked Immunosorbent Assay (ELISA) test for HIV. Three sets of ELISA tests were done according to NACO guidelines [15,16]. A sample testing reactive by the first test (SD BIOLINE HIV 1/2 ), underwent a second (PAREEKSHAK TRILINE HIV ½) and a third (PAREEKSHAK TRISPOT HIV 1/2 ) test based on different principles or different antigen systems. A sample testing reactive for all three tests was termed HIV seropositive. Once HIV status was confirmed, patients were registered in the ART clinic with permanent ART numbers. All patients underwent pre-registration assessment, consisting of complete history and physical examination with measurement of height, weight and body mass index (BMI). Laboratory investigations for each included plasma HIV RNA viral load and CD4 cell count; complete blood count (CBC), erythrocyte sedimentation rate (ESR), and serum biochemistry; smear for acid fast bacilli, culture and drug sensitivity for TB, and in some cases PCR of sputum or other specimens; diagnostic imaging like roentogram, ultrasonography, or computed tomography as indicated for TB diagnosis; histology or cytopathology of lymph nodes, abscesses or body fluids if indicated; routine urine and stool microscopy for evidence of other opportunistic infections; and testing for hepatitis B (HBsAg) and hepatitis C (anti-HCV IgG) infections.

As per the study protocol, all HIV-TB patients received ART regardless of CD4 cell count. Antiretroviral regimen consisted of stavudine (d4T) 30 mg or zidovudine (ZDV) 300 mg twice daily, along with lamivudine (3TC) 150 mg twice daily and efavirenz (EFV) 600 mg once daily. EFV was replaced with nevirapine at the end of TB treatment. Patients who developed ZDV- induced anaemia during the course of HAART had ZDV replaced with d4T. All the study participants who had CD4 cell count <200/mm3, were given trimethoprim-sufamethoxazole (TMP-SMZ), one double strength tablet once daily for Pneumocystis prophylaxis.

Since baseline visit at the initiation of HAART, patients from both the groups were followed every month till 12 months of ART. At follow up visits, each patient underwent a targeted history and physical examination, blood tests including serum biochemistry, CBC and ESR, and other relevant investigations based on clinical findings. CD4 cell counts were measured by flow cytometry using BD FACS CALIBUR and flourocein monoclonal antibodies (Beckton Dickenson Biosciences, California, USA) at baseline, two, six, nine, and 12 months of follow up. The HIV RNA viral load was measured by Roche Amplicor (Amplicor HIV-1 Monitor Test, version 1.5, Branchburg, NJ: Roche Diagnostics; 2003) at baseline, six, and twelve months. A sputum examination was done before the start of ATT, and at two and six months of ATT. Chest roentgenogram was done at baseline, at six months post ATT, and at the time of Immune Reconstitution Inflammatory syndrome (IRIS), if applicable. All patients coming for follow up visits were assessed for adverse events including IRIS, and the emergence of any opportunistic infections.

### Outcomes

The primary end points of the study were death from any cause and antiretroviral treatment failure, as a measure of progression of HIV disease. Death was documented from hospital death certificates, or by communication with the patient’s family or health care provider. As per NACO guidelines, a patient was said to have ART failure if any of the following occurred after at least six months of ART: - Clinical failure: new or recurrent WHO stage four condition; Immunological failure: a fall of CD4 count to below pre-therapy baseline, or a 50% decline in CD4 count from the on – treatment peak value, or persistent CD4 levels below 100 cells /mm^3^; virological failure: plasma viral load >10,000 copies/ml [17]. The secondary end points of the study were defined by safety and tolerability of ARV therapy, as assessed by the incidence of adverse events and proportion of subjects changing/ discontinuing ARV therapy because of the same. Outcome of TB treatment as defined by WHO [18], CD4 cell count and HIV RNA viral load, and assessment of general health by blood haemoglobin level and BMI at 6 and 12 months of ART were other secondary outcomes.

### Sample size

The null hypothesis of this study states that there is no difference in terms of all cause mortality and progression of HIV disease between HIV patients with tuberculosis who start HAART early (at 2-4 weeks) and those who receive it delayed (at 8-12 weeks) after commencement of antituberculosis chemotherapy. Since it was one of the first studies on this topic from India, a target sample size of 75 patients per arm was decided as the sample size of convenience as per protocol.

### Randomization

2 weeks after starting ATT, patients were allocated by computer generated sequence of random numbers to one of the two groups of the study in one to one ratio, following simple randomization. The sequence was generated by the statistician, and the study being open label, was known to the research team, which enrolled the participants as well as assigned them to study groups. ART physicians were informed of each patient’s allocation after randomization. Neither participants nor care providers were blinded to interventions.

### Statistical methods

All the pertinent clinical and laboratory data on individual subjects were entered into the case report forms, and transferred to an electronic data base with 100% retrospective data auditing. The electronic data was exported into the STATA software, version 11, for statistical analysis. All the analyses were performed as per the modified intention-to-treat principle with inclusion of only those patients who initiated HAART at the place of the study. The analyst was kept blind to the interventions. Primary end points of the study were assessed using Kaplan Meier analysis and log rank comparisons. Fisher’s exact test was used for analysis of categorical variables, and Student’s t-test for continuous variables.

## Results

### Participants

**Figure 1:**
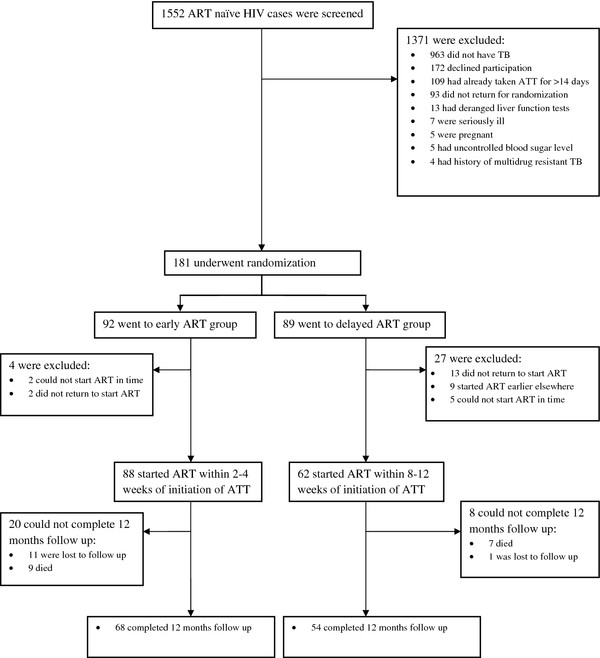
Screening, Enrolment, and Follow-up of study participants.

Figure 1 shows the screening and enrolment of participants in the study. A total of 181 patients were randomized, with 92 going to the early ART arm and 89 to the delayed ART arm. Fifteen of them (two from early ART and 13 from delayed ART groups) did not come back for HAART initiation, while seven others (two from early ART and five from delayed ART arms) could not start HAART before completion of their ATT. Besides, nine patients from the delayed ART arm started HAART earlier elsewhere and did not come for follow up. The remaining 150 patients were initiated on HAART as part of the study: 88 received it within 2-4 weeks (early group) of starting ATT, and 62 within 8-12 weeks (delayed group) of the same. At the time of the final analysis, 122 (81.3%) patients out of 150 had completed follow up of 12 months, 68 in early and 54 in delayed ART groups respectively. Sixteen (10.7%) patients, nine (10.2%) from early and seven (11.3%) from delayed ART group, had died, and 12 (8.0%) were lost to follow up, 11 (12.5%) in the early arm and one (1.6%) in the delayed arm. ‘Lost to follow up’ was defined by failure to visit the hospital for three consecutive months. Out of the total 12 patients lost to follow up, five were untraceable (no telephonic contact could be made), four were transferred to other ART centres due to spatial relocation, and three had defaulted treatment.

### Recruitment

All the patients enrolled till March 31, 2011, were included in the final analysis. The duration of follow up in the study was calculated as the time from initiation of HAART to death, loss to follow up, or the completion of 12 months whichever came earlier.

### Baseline data

Group-wise baseline demographic and clinical characteristic of 150 enrolled patients is presented in Table 1. Both the groups were found to be homogeneous with each other, having similar initial BMI, age distribution, and sex ratio (with significant male predominance). The median gaps between commencement of ATT and ART in early and delayed groups were 19 and 63 days respectively. No significant difference was seen between the groups in terms of baseline median CD4 count, viral load, and other laboratory parameters. Most patients were new cases of TB, and therefore, received DOTS category I ATT (94.3% in the early ART group, 98.4% in delayed ART group). According to RNTCP guidelines, they received isoniazid (H) 600 mg, rifampicin (R) 450 mg (600 mg if body weight ≥60 kg), pyrazinamide (Z) 1500 mg, and ethambutol (E) 1200 mg thrice weekly during the intensive treatment phase of two months, followed by isoniazid 300 mg and rifampicin 450 mg thrice weekly in the continuation phase for 4 months (2H_3_R_3_Z_3_E_3_ + 4H_3_R_3_). Six patients, who had been treated for a different episode of TB in the past, received DOTS category II ATT (five in early ART and one in delayed ART group). Intensive phase of RNTCP DOTS category II ATT lasts for three months, with streptomycin (S) 750 mg (500 mg if age >50 years) given thrice weekly for first two months, in addition to H_3_R_3_Z_3_E_3_. In continuation phase, category II patients received isoniazid, rifampicin, and ethambutol for 5 months (2H_3_R_3_Z_3_E_3_S_3_ + 1H_3_R_3_Z_3_E_3_ + 5H_3_R_3_E_3_) [5,6]. Even though the early ART group has apparently higher number of cases of EPTB with dissemination, no statistically significant difference was found between the two arms of the study with respect to any type of TB individually, or overall.

**Table 1:** Baseline Characteristics of the Study Participants

**Variable**	**Early therapy n = 88**	**Delayed therapy n = 62**	**P value**
Age, years			
Mean ± SD	34.86 ± 8.1	34.82 ± 7.6	0.97
Gender, number (%):			
Male	74 (84.1%)	52 (83.9%)	
Female	14 (15.9%)	10 (16.1%)	0.971
BMI, kg/m^2^:			
Mean ± SD	17.9 ± 2.8	18.4 ± 2.1	
Hemoglobin, mg/dl			
Mean ± SD	10.6 ± 1.7	11.0 ± 1.9	
CD4 count, cells/mm^3^:			
Median	133	152	
(Range)	(7 – 588)	( 14 – 648 )	
log_10_viral load/ml:			
Median	5.36	5.30	
(Range)	(2.42–5.88)	(2.33–5.88)	
Non TB OI’s :			
number (%)	12 (13.6%)	10 (16.1%)	0.671
WHO Staging of HIV disease, number (%):			
Stage 3	19(21.6%)	15 (24.2%)	0.70
Stage 4	69 (78.4%)	47 (75.8%)	(overall)
Type of Tuberculosis, number (%):			
PTB	19 (21.6%)	15 (24.2%)	
PTB with Dissemination	33 (37.5%)	26 (41.9%)	0.367
EPTB	18 (20.4%)	15 (24.2%)	(overall)
EPTB with Dissemination	18 (20.4%)	06 (09.7%)	
DOTS Category of ATT, number (%):			
Category I (2H_3_R_3_Z_3_E_3_ + 4H_3_R_3_)	83 (94.3%)	61 (98.4%)	0.214
Category II (2H_3_R_3_Z_3_E_3_S_3_ + 1H_3_R_3_Z_3_E_3_ + 5H_3_R_3_E_3_)	05 (5.7%)	01 (1.6%)	(overall)

SD = Standard deviation; BMI = Body Mass Index; OI’s = Opportunistic Infections; WHO = World Health Organization; HIV = Human immunodeficiency virus; PTB = Pulmonary Tuberculosis; EPTB = Extra Pulmonary Tuberculosis; ATT = Antituberculosis treatment; H = Isoniazid (600 mg), R = Rifampicin (450 mg), Z = Pyrazinamide (1500 mg), E = Ethambutol (1200 mg), S = Streptomycin (750 mg).

### Primary outcome

All 150 patients, 88 in the early ART group and 62 in the delayed ART group, were included in the analysis for primary outcome measures of death from any cause and ART failure. The overall mortality was found to be 10.7% (16/150), 10.2% (9/88) in the early ART group and 11.3% (7/62) in the delayed ART group (p value = 0.773). This translates to death rates of 13.8 and 13.0 per 100 person years for the two groups respectively. It was seen that out of 16 patients who died during the follow up, 11 (68.8%) had baseline CD4 value <100 cells/mm3, seven coming from the early and four from the delayed ART groups.

Table 2 shows the comparative outcome of antiretroviral treatment in the two groups of the study. The incidence of immunological failure was seen to be significantly higher at 22.6% (14/62) in the delayed ART group, in comparison with 9.1% (8/88) in the early ART group (p = 0.033). Only one patient of all 150 went into clinical failure, and he was from the delayed ART group (0.0% vs.1.6%, p = 0.413). Virological failure was documented in 8 patients from each group, its incidence being 9.1% in the early and 12.9% in the delayed ART groups (p = 0.592). Rate of overall treatment failure was observed to be almost double in the delayed ART group (31%), as compared to that in the early ART group (16%) (p = 0.045). The incidence of failure of ARV therapy in the entire study population was 22%.

**Table 2:** Outcomes of Antituberculosis and Antiretroviral Treatment

**Outcome of ATT at 6 months/completion**			
**Outcome**	**Early ART (n = 88)**	**Delayed ART (n = 62)**	**P value**
Successfully treated	93.4% (71/76)	93.1% (54/58)	0.105 (Overall)
Failure	6.6% (5/76)	5.2% (3/58)	
Treatment Modified	1.3% (1/76)	1.7% (1/58)	
**Losses during ATT:**			
Died	06 (6.8%)	04 (6.4%)	
Lost to Follow Up	06 (6.8%)	00 (0.0%)	
**Outcome of ART at 12 months**			
Outcome	Early ART (n = 88)	Delayed ART (n = 62)	P value
HIV Disease Progression	14 (16%)	19 (31%)	0.045
Clinical Failure	00 (0%)	01 (1.6%)	0.413
Immunological failure	08 (9.1%)	14 (22.6%)	0.033
Virological failure	08 (09.1%)	08 (12.9%)	0.592
Lost to follow up	11 (12.5%)	01 (1.6%)	

ART = Antiretroviral therapy, ATT = Antituberculosis treatment.

**Figure 2:**
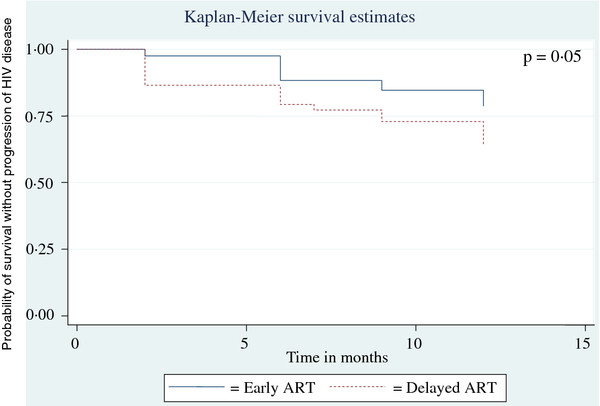
Kaplan Meier Curve of Survival without progression of HIV disease for 12 Months with Early and Delayed ART.

Based on the incidence of various types of treatment failure over the follow up period, 12 months HIV disease progression free survival rate was calculated for each group. At 79%, it was significantly better for the early ART group, as against 64% for the delayed ART group (p = 0.05). A Kaplan-Meier disease progression free survival curve was plotted on the basis of the same calculation and is depicted in Figure 2.

### Secondary outcomes

Among the adverse events, immune reconstitution inflammatory syndrome was diagnosed in nine patients (10.2%) in the early ART group and six patients (9.7%) in the delayed ART group (p = 0.571). The median time of occurrence of IRIS after initiation of HAART was 73 and 68.5 days in the early and delayed ART groups respectively. All cases of IRIS were of mild to moderate severity, and none of them required any interruption of HAART or management with steroids. The affected patients responded well to antipyretic (paracetamol) and analgesics (non-steroidal anti-inflammatory drugs).

The incidence of other adverse events was also not significantly different between the groups (23.9% in the early and 22.6% in the delayed ART group, p = 0.855). Seven patients (4%) required a change in the antiretroviral regimen, in which zidovudine was switched for stavudine, due to the development of drug induced severe anaemia. Four (4.5%) of them came from the early ART group and three (4.8%) from the delayed ART group (p = 0.612). No change in therapy was needed for any other adverse event. All of them were managed appropriately by experts at the respective follow up clinics of the patients. No death in the study population was attributed to any of the adverse events.

Outcome of antituberculosis treatment was categorised as per the WHO definitions [19]. At the time of final analysis, all study participants had completed their prescribed ATT courses. Six (6.8%) patients from early and four (6.4%) from delayed ART group had died, and further six patients from the early ART group were lost to follow up before the completion of their antituberculosis therapy. No significant difference was seen in the outcome of TB treatment at the completion of ATT between the groups (p = 0.105), as summarized in Table 2. During follow up, one case of relapse of TB has been reported from each group till date.

Response of the patients to overall treatment was similar in each group in terms of general health parameters like haemoglobin level, liver function tests and body mass index (Table 3).

**Table 3:** Health Parameters at Different Time Points

**Variable↓ Group → Time →**	**Early ART**			**Delayed ART**		
	**Baseline**	**6 months**	**12 months**	**Baseline**	**6 months**	**12 months**
Bilirubin, mg/dL: Mean ± SD	0.6 ± 0.2	0.7 ± 0.1	0.7 ± 0.1	0.7 ± 0.1	0.7 ± 0.1	0.7 ± 0.1
SGOT, IU/L: Mean ± SD	51.1 ± 29.6	48.3 ± 20.8	35.8 ± 17.1	47.6 ± 20.9	40.2 ± 19.8	33.6 ± 9.9
SGPT, IU/L: Mean ± SD	39.1 ± 23.7	42.4 ± 19.4	31.1 ± 11.6	40.3 ± 20.2	34.1 ± 14.1	33.2 ± 17.7
Hemoglobin, g/dL: Mean ± SD	10.2 ± 1.8	12.2 ± 1.9	12.8 ± 1.3	10.6 ± 1.8	12.3 ± 1.5	12.8 ± 1.4
ESR, mm/1^st^ hour: Mean ± SD	46.0 ± 23.8	28.7 ± 17.2	25.7 ± 11.5	43.0 ± 24.6	32.4 ± 15.2	22.0 ± 9.9
BMI, kg/m^2^: Mean ± SD	17.9 ± 2.8	19.9 ± 2.5	20.7 ± 2.7	18.4 ± 2.1	20.2 ± 2.4	20.9 ± 2.5

ART = Antiretroviral therapy; SD = Standard deviation; SGOT = Serum Glutamate Oxaloacetate Transaminase; SGPT = Serum Glutamate Pyruvate Transaminase. ESR = Erythrocyte Sedimentation Rate; BMI = Body mass index.

## Discussion

This is the first study sponsored by National AIDS Control Organization, Ministry of Health & Family Welfare, Government of India in this topic. The results of this study show that once highly active antiretroviral therapy (HAART) is started, there is no significant difference in mortality between HIV-TB patients who were started on HAART 2-4 weeks after initiation of antituberculosis treatment (early ART group), and those who received it 8-12 weeks after starting ATT (delayed ART group). However, the risk for progression of HIV disease, as estimated by onset of failure of ARV therapy, increases significantly if initiation of HAART is deferred from 2-4 weeks to 8-12 weeks (16% vs. 31% risk, p = 0.045). The incidence of IRIS and other adverse events, and outcome of TB treatment are similar between the groups.

To decide the optimal timing for initiation of HAART after starting ATT in HIV-TB patients has been listed as a priority research question for resource limited settings by WHO [20]. Several studies have been involved with the task of finding a definitive answer to it so that mortality and serious morbidity could be minimized in this patient population [12,21]. A recent trial by Abdool Karim *et al* shows that starting antiretroviral therapy at CD4 cell count <500/mm^3^ during treatment for AFB smear-positive TB (integrated therapy) reduces mortality by 56% in HIV-TB cases, as compared to delaying it until the completion of TB therapy (sequential therapy) [22]. They have reported an all-cause mortality rate of 5.4 per 100 person-years in the integrated therapy arm as compared to 12.1/100 per year in the sequential therapy arm (p = 0.003). Their integrated therapy arm corresponds roughly with both the groups of the present study taken together, where the combined mortality rate was observed to be 13.4/100 per year. The main reason for this apparently higher mortality observed here may be that the current study had a much higher percentage (77.3%) of patients in WHO stage four HIV disease at baseline than that (4.9%) in the South African study.

Another trial at Cambodia has shown in its preliminary report that there is a significant survival advantage of 34% in starting HAART at two weeks (early arm) rather than at eight weeks (late arm) after initiation of TB treatment [19,23]. They observed a mortality of 17.8% in the early arm against 27.4% in the late arm (p = 0.002). On comparison, it can be seen that the Cambodian study reports a much higher mortality rate in both the groups than the present study despite both following a similar protocol in terms of timing of ART initiation. One of the important reasons for this difference may be their exclusive enrolment of patients with CD4 count <200/mm^3^. For the same reason, it can be argued that results of the current study are more generalizable than the Cambodian one. Moreover, while the present research work has excluded the deaths that might have occurred between the starting of ATT and ART in both the groups, this fraction was taken into account while calculating mortality in the Cambodian project.

The current research was designed to assess and compare the response of HAART in HIV-TB patients who started it 2-4 weeks after starting TB therapy and those who received it 8-12 weeks after starting TB treatment. CD4 cell counts and plasma viral load have been considered important markers for assessment of response to ARV therapy and disease progression [24-26]. Previous studies have shown that co-infection with TB results in reduced survival, increased risk for other opportunistic infections and elevations in HIV replication [27,28]. Increased HIV replication is attributed to activation of latently infected cells, and promotion of infection in uninfected lymphocytes and macrophages. HIV genetic diversity is also increased in the presence of active TB infection [29-31]. The results of this study indicate that these processes may be significant enough to cause failure of ARV therapy after a few months, warranting a switch to second line ART. Some previous studies have demonstrated that death in HIV-TB patients within the first few months of TB treatment may be related to TB, whereas late deaths are attributable to HIV disease progression [32-34]. Therefore, it can be extrapolated that in due course of time a differential disease progression might even translate into a significant difference in mortality between the two groups of the study. In light of this observation, the present work supports 2010 WHO recommendations for the management of HIV-TB cases: [35]

1. Start ART in all HIV-infected individuals with active TB, irrespective of the CD4 cell count.

2. Start TB treatment first, followed by ART as soon as possible afterwards (and within the first eight weeks).

The overall incidence of Immune Reconstitution Inflammatory Syndrome observed here (10.0%) is close to that reported for the integrated therapy arm (12.4%) by Abdool Karim *et al*[27]. The Cambodian trial has reported a significantly higher incidence of IRIS in the early ART arm (4.03 per 100 patient months) than in the late arm (1.44/100 pm, p <0.0001) [28], but no such difference could be seen in the present study.

This research work has a few limitations. Here all cause mortality was taken as the primary outcome instead of disease-specific death rate. While the latter would be more suitable for assessment of response to ART, there were logistic difficulties in ascertaining the exact cause of most of the deaths, as they happened away from the hospital. Another limitation is introduced by the uneven number of lost to follow up patients (11 in early ART and one in delayed ART) in the two groups of the study, which could affect its results. But seven of them from early ART arm were known to be alive by the end of 12 months since they were initiated on ART, and the consideration of the worst outcome for the five (four in early ART arm and one in delayed ART arm) untraceable patients did not alter the overall results of the study significantly. In addition, as decided in the approved protocol, sample size of the study was based on a target of convenience rather than on power calculations. In this study given a baseline rate of 10% mortality, there was an 80% power to detect a difference of 18% mortality between early and delayed ART groups. However, despite these limitations, being one of the first studies on this topic from India, the present work will serve as one of the resources for early starting of ARV treatment under National AIDS Control Programme. It will also help for any future metaanalysis for the timing of HAART initiation in HIV-TB cases.

This study presents important research findings regarding optimal time to initiate ART in HIV-TB cases so that detrimental effects of both the diseases can be minimized. Since it included HIV patients irrespective of baseline CD4 cell counts and with different types of TB, its results can be fairly generalized. It brought to light the observation that starting early ART in HIV-TB patients helps control the progression of HIV disease later on, and hence, it suggests that all such patients must be started on HAART as soon as possible after initiation of TB treatment.

## Competing interest

We declare that we have no conflicts of interest.

## Authors’ contributions

SS provided inputs to the study design, helped in data analysis and interpretation, wrote the manuscript, and did final editing. SRC, SG, EM and SN reviewed literature, and helped in interpreting data and writing the manuscript. AH and KN collected data, and conducted laboratory tests for CD4 cell count and plasma viral load. RS helped in data collection. SV did data analysis. SSK, SJC, and MRT edited the manuscript. All authors read and approved the final manuscript.

### Funding

National AIDS Control Organization (NACO), Ministry of Health & Family Welfare, Government of India, New Delhi, India.

## Pre-publication history

The pre-publication history for this paper can be accessed here:

http://www.biomedcentral.com/1471-2334/12/168/prepub
